# Screening and Activity Evaluation of Peptides With Anti‐Ice‐Nucleation Activity Using the Phage Display Library Method

**DOI:** 10.1002/psc.70141

**Published:** 2026-10-22

**Authors:** Sora Okamoto, Seijiro Matsuki, Yoshiaki Hirano

**Affiliations:** ^1^ Department of Chemistry and Materials Engineering, Faculty of Chemistry Materials and Bioengineering Kansai University Suita Japan

## Abstract

We screened for anti‐ice‐nucleation peptides using the phage display library method, with silver iodide as the target for artificial ice nucleation The phage interacted with the silver iodide in a dispersed state, rather than in an immobilized state. Based on DNA analysis, we identified five peptides with high expression levels. Five peptide sequences were synthesized by hand using the Fmoc solid‐phase peptide synthesis and evaluated their anti‐ice‐nucleation activity. Three peptide sequences exhibited anti‐ice‐nucleation activity, and two of them were insoluble in water. Among the three peptides, SHIARSV exhibited the highest anti‐ice‐nucleation activity. To determine the importance of each amino acid in SHIARSV, we designed five peptides in which Ala was substituted and examined their anti‐ice‐nucleation activity. As a result, we identified AHIARSV, a peptide sequence with higher anti‐ice‐nucleation activity than the original SHIARSV sequence. This peptide exhibited high levels of both anti‐nucleation activity and ice recrystallization inhibition activity. It is our conclusion that the design of antifreeze peptides with activities comparable with those of natural ones is highly feasible.

## Graphical abstract

AHIARSV, a peptide sequence with higher anti‐ice‐nucleation activity than the original SHIARSV sequence, exhibits high levels of both anti‐nucleation activity and ice recrystallization inhibition activity.

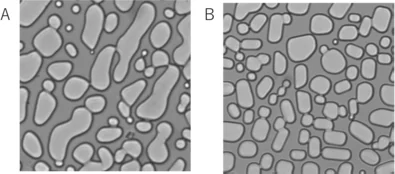

## 1 Introduction

The phage display method, a genetic engineering technique, is used to create useful peptides and proteins that do not occur naturally [1]. Among the various phage display methods, the surface‐displayed peptide library allows random peptides to be presented on the surface of phages. This method enables the efficient identification of peptides that exhibit binding affinity for target molecules or substrates. Smith et al. made it possible to display peptides with random amino acid sequences on the surfaces of filamentous phages and, when necessary, to propagate them in *E. coli* [1].

In recent years, screening for affinity ligands using peptide libraries has led to the identification of peptides that exhibit binding affinity for a wide variety of targets, including monoclonal and polyclonal antibodies, protein receptors, enzymes, small molecules, cells, and plastics [2, 3, 4, 5]. In this study, we focused on anti‐ice‐nucleus substances that exhibit binding affinity for ice nuclei. One example is antifreeze protein (AFP), which is produced within plants and organisms living in low‐temperature environments [6, 7, 8]. Generally, AFP adsorbs onto ice nuclei and controls the shape and size of ice crystals by inhibiting their growth. However, because AFP is a protein, it is difficult to synthesize artificially; furthermore, due to its rarity, extracting it from living organisms is likely to be challenging [9, 10].

AFP exhibits two main types of antifreeze activity. The first is thermal hysteresis (TH) activity [11, 12, 13, 14, 15], which lowers only the freezing temperature without changing the melting temperature. TH activity is thought to occur when AFP adsorbs onto the basal or prismatic faces of ice crystals [9], and it is known that adsorption onto the ice crystal surface depends on the AFP concentration. Although disc‐shaped ice crystal growth is observed in solutions without AFP, in the presence of low concentrations of AFP, the prismatic faces undergo weak growth inhibition due to AFP adsorption, resulting in the formation of hexagonal planar ice crystals [10]. In the presence of high concentrations of AFP, crystal growth along the *a*‐axis is almost completely suppressed, resulting in growth only along the *c*‐axis and forming a slender hexagonal bipyramidal shape [16]. This function allows organisms to maintain their vital functions even in low‐temperature environments without freezing their own cells [17].

The second is ice recrystallization inhibition (IRI) activity [18, 19]. Ice recrystallization is a phenomenon in which ice crystals grow within the temperature range of 0°C to −20°C; it is believed to be caused by the aggregation of ice crystals or by Ostwald ripening, in which small ice particles melt into water, adsorb onto the surface of larger ice crystals, and cause them to grow larger. Therefore, when ice crystals come into contact with one another, the phenomena mentioned above occur, manifesting as the recrystallization phenomenon [19, 20]. AFP suppresses this ice recrystallization by adsorbing onto the surface of ice crystals. Through this function, it inhibits ice growth and prevents cell damage [17].

Consequently, we considered using peptides as an alternative and attempted to screen for peptides with binding affinity for ice nuclei using the phage display method. However, because it is difficult to use dust or metal ions—which act as ice nuclei—as target molecules, we aimed to establish a biopanning method using AgI, which is employed in artificial rainfall [21, 22, 23, 24] and has a structure similar to that of ice crystals, as a model ice crystal.

In this study, we searched for peptides with anti‐ice‐nucleation activity (ANA) [8, 25, 26, 27, 28]. We synthesized the identified peptide sequences and evaluated their anti‐ice‐nucleation activity using the microdroplet freezing method [29, 30, 31, 32, 33]. Furthermore, we identified the effective amino acids in the peptide that exhibited the highest anti‐ice‐nucleation activity through an alanine scan, measured IRI activity [8, 18, 19, 34] using the sucrose sandwich method [20, 35], and evaluated the structure of the ice crystals to determine whether the peptide possesses anti‐ice‐nucleation activity as an alternative to AFP.

## 2 Material and Method

### 2.1 Toxicity Assessment of AgI on *E. coli*

Prior to performing biopanning using AgI, we first tested the toxicity of AgI on *E. coli* under light‐shielded conditions. In culture medium containing only *E. coli* , the cells spread without any gaps. In contrast, in culture medium containing AgI, although the spread was slightly less extensive than in the *E. coli* ‐only medium, the *E. coli* cells did not die and were still able to grow; therefore, it is considered that AgI is not toxic to *E. coli* .

### 2.2 Biopanning [36, 37, 38, 39, 40, 41]

A Ph.D‐7 Phage Display Peptide Library Kit, a Ph.D. Peptide Display Cloning System, and *Escherichia coli* strain K12 ER2738 were purchased from New England Biolabs (Ipswich, MA, United States). Ph.D.‐7 Phage Display Peptide Library was containing 1.0 × 10^13^ plaque‐forming units (pfu) phage solution in 1 mL of Tris‐buffered saline (TBS; 50 mM Tris, 150 mM NaCl, pH 7.5). The phage‐based targeting was performed using a method that did not involve immobilizing AgI on a substrate. For the AgI dispersion, 5 μL of phage solution diluted 1000‐fold in TBS‐T buffer was added, and the mixture was shaken. See Table 1 for all numerical arrangements. The interacted solution was centrifuge. The supernatant was removed, and the precipitated AgI was resuspended in 5 mL of TBS buffer. To cleave the disulfide bonds in the phage tail, 12 mL of glycine‐HCl buffer (pH 2.2) was added, and the mixture was shaken. The mixture was neutralized by adding 2 mL of Tris–HCl buffer. Centrifugation was performed to remove the AgI, and the entire supernatant was recovered (E1. Solution).

**TABLE 1:** Biopanning condition of each round.

	Amount of AgI/10 ml TBS	Interaction time	Number of washes	Wash time
Round 1	10 mg	1 h	1	1 min
Round 2	10 mg	1 h	3	1 min
Round 3	10 mg	1 h	3	15 min
Round 4	10 mg	1 h	3	15 min

To culture *E. coli* for phage infection, a small amount of *E. coli* was scraped into 5 mL of LB/Tet medium and incubated. We diluted the E1 solution with LB/Tet medium to a dilution range of 10^1^ to 10 [4]. We added 20 μL of the undiluted E1 solution and 20 μL of each dilution (10^1^ to 10 [4]) to 180 μL of the *E. coli* culture, separately, and allowed them to stand. For each sample, the infection solution was vortexed to homogenize the phages in the solution; then, 10 μL portions were seeded onto the agar plates and incubated. The control consisted of Top Agar medium to which only an *E. coli* culture was added. After infection, the contents of the baffle flask were transferred to a centrifuge tube and the amplified phages were then purified using a polyethylene glycol/NaCl solution, and the supernatant was collected. An 80% glycerol solution was added and mixed by pipetting. This mixture solution was done for use in the next stage of peptide screening.

For Round 2 and beyond, the experiments were conducted following the same procedure as in Round 1 and in accordance with the conditions shown in Table 1.

### 2.3 DNA Analysis

Add 1 mL of phage lysate from Rounds 3 and 4 (1.0 × 10^12^ pfu/mL) to a 2.0‐mL tube. Add 500 μL of lysis buffer B and vortex for 10 s. Incubate at 65°C for 15 min, and add 320 μL of isopropanol to the lysate and vortex. Add 650 μL of lysate to the column, centrifuge (room temperature, 6600 rpm, 1 min), and remove the supernatant; repeat this step until no lysate remains.

Wash solution A (containing ethanol) was added to the column, followed by centrifugation (room temperature, 6600 rpm, 1 min) to remove the solution; this procedure was repeated three times. For the final steps, add 75 μL of Elution Buffer B to the column, centrifuge (room temperature, 6600 rpm, 5 min), and collect the solution. Measure the nucleic acid concentration based on absorbance using BioSpec‐nano (SHIMADZU BIOTECH, Kyoto, Japan). DNA analysis was performed using amplicon sequencing at Bioengineering Lab. Co. Ltd. (Sagamihara, Kanagawa, Japan).

### 2.4 Peptide Synthesis [42, 43]

All 9‐fluorenylmethyloxycarbonyl (Fmoc) amino acid derivatives and solid phase peptide synthesis resins were purchased from Watanabe Chemical Industries Ltd. (Hiroshima, Japan). All other reagents including organic solvents were purchased from FUJIFILM WakoPure Chemical Corporation (Osaka, Japan). We synthesized seven‐residue peptides from the top five types selected through biopanning. The top five peptide and related peptides that induce the cell aggregate were synthesized by the solid phase peptide synthesis procedure. The related peptides were synthesized on Alko‐PEG resin using a handmade standard manual Fmoc‐protocol with a 4‐(4,6‐dimethoxy‐1,3,5‐triazin‐2‐yl)‐4‐methylmorpholinium chloride (DMT‐MM) activation procedure [44, 45]. *N*‐(9‐Fluorenylmethoxycarbonyl)‐L‐Xaa‐tritylcarboxamidomethyl polyethylene glycol resin (Fmoc‐Xaa‐Alko‐PEG‐Resin (0.20 mmol) was placed in a polypropylene column (PD‐10 column, empty, Merck and Co. Inc., Rahway, NJ, United States) and washed three times with *N*,*N*‐dimethylformamide (DMF) and methanol. The resin was swollen with 25% (w/v) dimethyl sulfoxide (DMSO)/DMF for 30 min. The mixture was then reacted with 20(w/v) piperidine (PPD)/DMF for 30 min to deprotect the Fmoc group. Then, 0.6 mmol Fmoc‐protecting amino acid, 0.06 mmol *N*‐methylmorphiline, and 1.2 mmol DMT‐MM were added to the resin. The condensation reaction was run for 120 min. The resin was washed six times with DMF for 1 min each time. The Fmoc group deprotection and condensation reactions were repeated to synthesize protected peptide‐Alko‐PEG Resin. Finally, peptide protecting group deprotection and resin cleavage were conducted under acidic conditions. The protected peptide‐Alko‐PEG‐resin and the cleavage mixture (8.50 mL trifluoroacetic acid [TFA], 0.50 mL thioanisole, 0.80 mL pure water, 0.25 mL 1,2‐ethanedithiol, and 0.75 g phenol) were stirred for 150 min [42, 43]. Then, diethylether was added to reaction mixture, and the precipitate was filtered and dissolved in 50 mL of water. The crude peptides containing solution was dialyzed with a membrane with a molecular weight cutoff range of 100–500 Da (Spectra/Por Dialysis Membrane Biotech CE Tubing, MWCO: 100–500D). Other peptides were also synthesized in the same manner as above. Finally, all peptides were purified by high‐performance liquid chromatography (HPLC 8020 System; Tosoh Corp., Tokyo, Column: TSKgel‐ODS‐100 V 5 m) with a gradient of water/acetonitrile containing 0.1% TFA (20%–90% water/acetonitrile), under the following condition: flow: 1.0 mL/min, UV absorption wavelength: 210 nm, column oven temperature: 40°C. All peptides were identified by matrix‐assisted laser desorption/ionization mass spectrometry (MALDI‐TOF MS; Microflex LRF System, Bruker Corp., Billerica, MA, United States) using 4‐cyano‐4‐hydroxycinnamic acid as a matrix (Sigma‐Aldrich Co. LLC, Saint Louis, MO, United States).

### 2.5 Secondary Structure Analysis by Circular Dichroism (CD) Spectrum

Secondary structure analysis was performed using a CD dispersometer (J‐1500: JASCO Corporation, Japan). The peptide samples were prepared by mixing a 200‐μM aqueous peptide solution. The measurement was performed at 25°C (measurement wavelength of 190 to 250 nm, a data interval of 1 nm, a scanning speed of 100 nm/min, a cell length of 0.1 cm, and six integrations).

### 2.6 Measurement of Anti‐Nucleation Activity (ANA) Assay

Preparation of an AgI aqueous solution as a pseudo‐ice nucleus: AgI and potassium phosphate buffer (KPB) were mixed to a concentration of 1.0 mg/mL to prepare an AgI aqueous solution. The AgI aqueous solution was mixed with ultrapure water in a 9:1 ratio. AgI was used as a simulated ice nucleate.

The microdroplet freezing method described by Vali et al. was used to measure anti‐ice‐nucleation activity [29]. An aluminum plate was placed inside the CryoPorter, a programmable ultra‐low‐temperature aluminum block thermostatic chamber (manufactured by Sainix Co. Ltd.). Twelve 10‐μL droplets of the AgI aqueous solution were dispensed onto the surface of the aluminum plate, and the temperature of the cold plate was lowered at a rate of 1.0°C/min. The temperature (T50) at which six of the 12 droplets froze was recorded as the sample's freezing temperature (×3 times). Each sample was measured three times, and the ANA was calculated as: Sample (T50) − Control (T50).

For each sample, the peptide was dissolved in an AgI aqueous solution to concentrations of 1.0, 2.0, and 4.0 mg/mL. An AgI aqueous solution was used as the control.

### 2.7 Measurement of IRI Activity

To observe ice crystals formed when an aqueous solution containing peptides was frozen, IRI activity was measured using the sucrose sandwich method1 [8, 19]. A microscope equipped with a temperature control device (manufactured by Linkam) was used. Samples were prepared by dissolving the synthesized screening peptides in ultrapure water to a concentration of 1 mg/mL each. Each sample was mixed with a 60% (w/v) sucrose aqueous solution in a 1:1 ratio, and 1 μL of the mixed solution was sandwiched between two cover slips. The cover slips were placed on the temperature‐controlled device and rapidly frozen at a rate of 100°C/min down to −40°C. The temperature was then immediately raised to −6°C at the same rate. Ice crystals were photographed 30 min after reaching −6°C. The captured image was cropped to a 500 × 500 size, and the number of ice crystals and the total area of the ice crystals within that region were determined to calculate the average size per ice crystal (unit crystal area). Based on the measured size per unit area, IRI activity was calculated using the following equation. Note that a lower IRI value indicates higher activity. The control solution consisted solely of ultrapure water [18].

IRI value = Unit crystal area of the sample solution/Unit crystal area of the control solution.

## 3 Results and Discussion

### 3.1 Result of DNA Analysis

Using linear phages and the conditions described in Table 1, we performed four sequential rounds of biopanning. In Round 4, we extracted the DNA and measured the nucleic acid concentration; because it had reached the required level, we proceeded with DNA analysis. The peptides screened by biopanning are shown in Table 2.

**TABLE 2:** Peptide sequences identified by biopanning.

Peptide sequence	Counts
RPAVYHH	38
SHIARSV	37
GASTRVQ	27
GAPNAVP	19
QLVTGPS	18

### 3.2 Peptide Synthesis

Peptides with ANA in Table 2 were synthesized manually by solid phase peptide synthesis. All peptides were characterized by HPLC, MALDI‐TOF MS, and amino acid analysis. All peptides were purified by RT‐HPLC to a single peak. The results of MALDI‐TOF MS spectra m/z of [M + 1]^+^, [M + Na]^+^, and [M + K]^+^ were shown in Table 3. These results suggest that all peptides were synthesized as intended in the molecular design.

**TABLE 3:** Results of MALDI‐TOF MS spectra analysis of synthetic peptides.

Peptide sequences		Date of MALDI‐TOF MS		
	Fw (Cal.)	[M]^+^, [M + 1]^+^	[M + Na]^+^	[M + K]^+^
RPAVYHH (RP)	879.00	880.1	—	902.0
SHIARSV (SH)	768.89	769.7	—	—
AHIARSV	752.89	752.8	774.7	—
SAIARSV	702.82	702.8	724.7	740.6
SHAARSV	726.81	726.9	748.8	764.8
SHIAASV	683.77	683.6	705.7	721.3
SHIARAV	752.89	752.2	774.1	789.9
SHIARSA	740.84	740.3	762.3	778.2

### 3.3 Secondary Structure Analysis by CD Spectrum

All samples' HT values in the CD measurement were less than 500 in 200–250 nm. In 195–200 nm, HT values were less than 600.

Spectral measurements revealed that, in dilute solution, they exhibited a spectral pattern characterized by a negative maximum near 190–200 nm. The eight types of peptides shown in Table 3 formed random coil structures.

Because the peptide chain is short at seven residues, it is thought to be difficult for it to maintain a stable secondary structure in solution. Because of its random coil structure, it is thought that the peptide adsorbs randomly onto the surface of the ice nuclei and inhibits ice growth.

### 3.4 ANA Assay

The ANA values for the three synthesized compounds are shown in Table 4.

**TABLE 4:** Anti‐nucleation activity of top five peptides.

Peptide sequence (*abbreviation*)	ANA value
RPAVYHH (RP)	3.67 ± 0.31
SHIARSV (SH)	4.80 ± 0.60
GASTRVQ	Insoluble in water
GAPNAVP	Insoluble in water
QLVTGPS	Poorly soluble in water

SHIARSV exhibited the highest ANA. GASTRVQ and GAPNAVP could not be assessed because they are insoluble in water. QLVTGPS sequence was poorly soluble in water. Therefore, these three types of ANA could not be measured. An alanine scan was performed on the amino acid sequence of the SHIARSV (SH) peptide to identify the amino acids that influence its binding affinity for AgI [46].

We measured the anti‐ice‐nucleation activity of the synthesized peptides RPAVYHH, SHIARSV AHIARSV, SAIARSV, SHAARSV, SHIAASV, SHIARAV, and SHIARSA. We also examined the concentration dependence of these peptides by measuring their activity at concentrations of 1.0, 2.0, and 4.0 mg/mL. The results are shown in Figure 1. Because no concentration‐dependent response was observed, it was determined that the anti‐ice‐nucleation activity is not due to freezing point depression.

**FIGURE 1:**
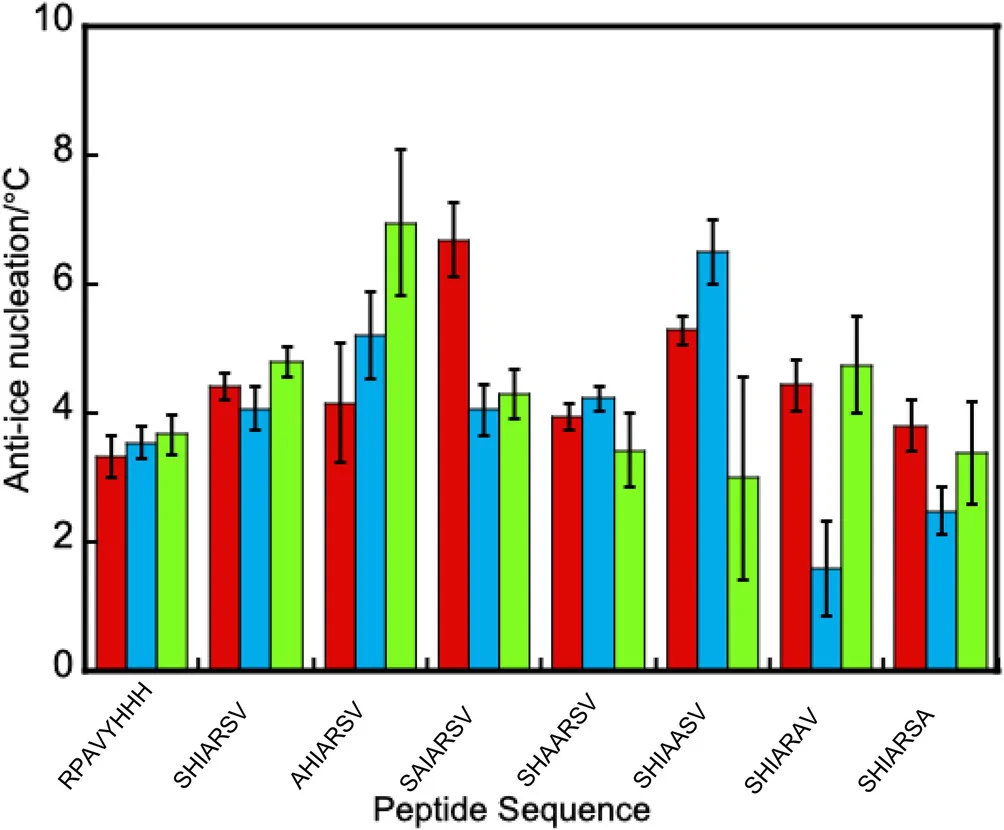
Anti‐ice nucleation activity values of RP and SH peptide analogs. Peptide concentration ■: 1 mg/mL, ■: 2 mg/mL, ■: 4 mg/mL. The peptide was dissolved in an AgI aqueous solution to concentrations of 1, 2, and 4 mg/mL.

In SHIARSV, in which the Ser at the first residue of the SH peptide was replaced with Ala, the anti‐ice‐nucleation activity was the same as that of the SH peptide up to 1.0 mg/mL, but it increased significantly at 2.0 and 4.0 mg/mL. To determine whether the anti‐ice‐nucleation activity was concentration‐dependent, we measured the activity at a concentration of 8.0 mg/mL and found that the value dropped sharply to 3.3°C. Generally, freezing point depression occurs in proportion to concentration. However, considering the decrease in activity observed at 8.0 mg/mL, it became clear that at 2.0 and 4.0 mg/mL, this peptide not only lowers the freezing point but also exhibits anti‐ice‐nucleation activity.

In SAIARSV, in which the second histidine residue of the SH peptide was replaced with alanine, the highest activity was observed at 1.0 mg/mL, and the anti‐ice‐nucleation activity was significantly reduced. At concentrations of 2.0 and 4.0 mg/mL, the values were essentially the same as those for the SH peptide.

For SHAARSV, in which the third residue (Ile) of the SH peptide was replaced with Ala, there was no change in the anti‐ice‐nucleation activity values compared with the SH peptide at any concentration. As for SHIAASV, in which the Arg at the fifth residue of the SH peptide was replaced with Ala, the activity increased significantly at 1.0 and 2.0 mg/mL. However, it decreased significantly at 4.0 mg/mL.

For SHIARAV, in which the Ser at the sixth residue of the SH peptide was replaced with Ala, the values were almost identical to those of the SH peptide at 1.0 and 4.0 mg/mL, but the anti‐ice‐nucleation activity values decreased significantly at 2.0 mg/mL. For SHIARSA, in which the Val at the seventh residue of the SH peptide was replaced with Ala, the values were almost identical to those of the SH peptide at 1.0 to 4.0 mg/mL, but the anti‐ice‐nucleation activity decreased at 2.0 mg/mL. It is unclear exactly why the concentrations of these two types of peptides (2 mg/mL) were the only ones to decrease. Because this result was reproducible, it is presumed that the interaction between the silver iodide ice nuclei and the peptides had some effect. Furthermore, because the results differed even though the same Ser residue was substituted, it is thought that the balance between hydrophobicity and hydrophilicity, which depends on the position of Ser within the peptide, is important.

I. K. Voets and co‐worker reported that upon dissolution of the peptide, nanoscale self‐assembled aggregates were observed to form in a pH‐ and concentration‐dependent manner. Light scattering (LS) measurements revealed the presence of both large and small aggregates, whereas cryo‐transmission electron microscopy (cryo‐TEM) observations revealed the presence of one‐dimensional structures (bundle‐like aggregates) [47].

This self‐organization behavior is prominently reflected in IRI activity. This is thought to be due to the temporary inactivation of ice‐binding sites caused by bundle‐like aggregation in a pH‐ and concentration‐dependent manner.

Although most natural ice‐binding proteins are rigid, artificial molecular ice‐binding proteins are typically flexible. Therefore, achieving a regular arrangement with ice‐binding motifs precisely positioned on synthetic proteins is a difficult challenge for (high)molecular ice‐binding proteins [48].

Based on our results, it can be inferred that a similar phenomenon is occurring, explaining why ANA activity values change dramatically in SHIARAV and SHIARSA only at specific concentrations.

### 3.5 Ice Recrystallization Inhibition Activity

The result photographs of IRI activity measurements for the synthesized RP peptide, SH peptide, AHIARSV, SAIARSV, SHAARSV, SHIAASV, SHIARAV, and SHIARSA are shown in Figure 2, whereas Figure 3 shows the IRI activity of each peptide [49].

**FIGURE 2:**
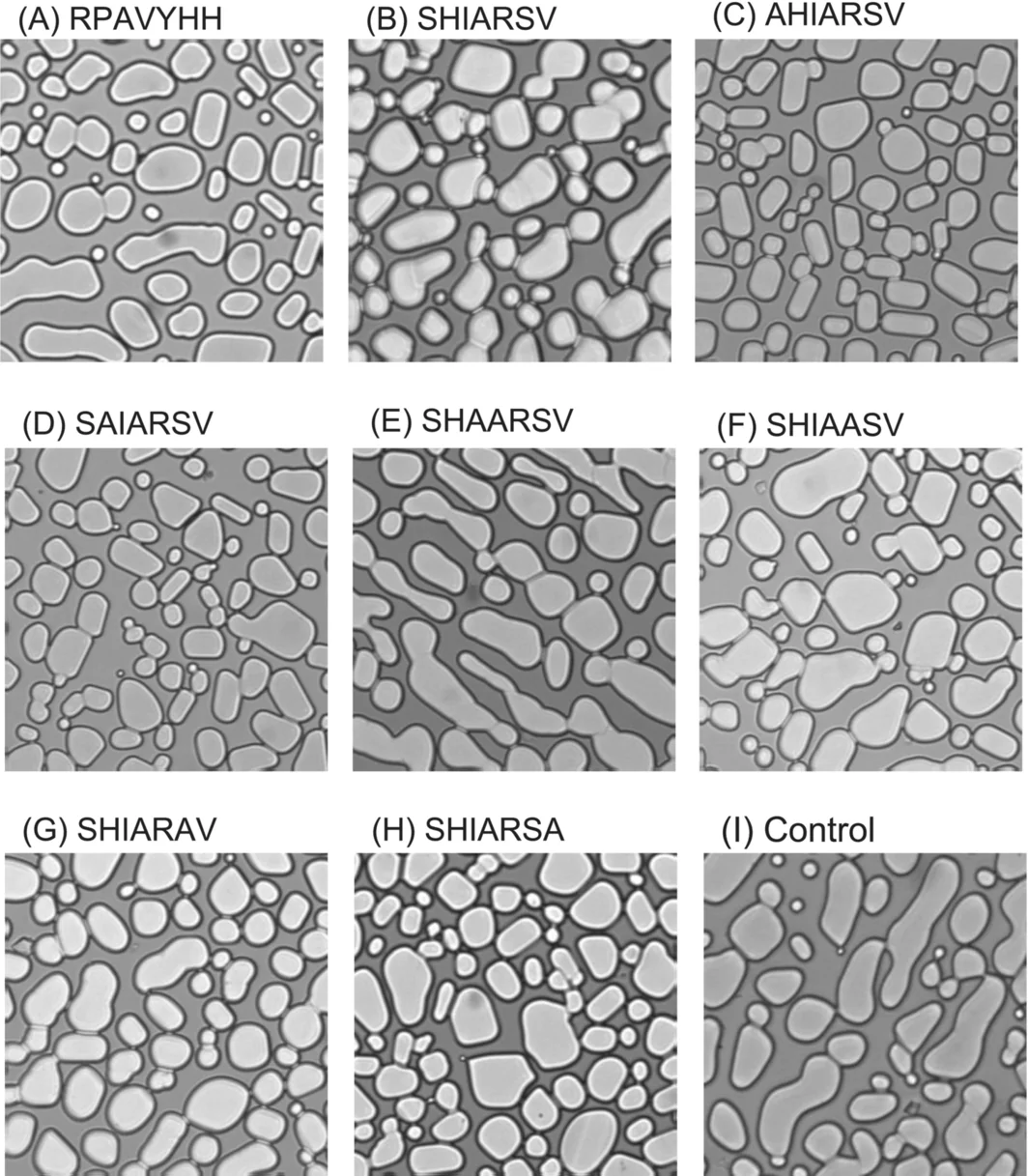
Ice recrystallization inhibition activity images of RP and SH peptide analogs (30 min later). The formation of ice crystals with a small surface area and angular shapes indicates high reactivity.

**FIGURE 3:**
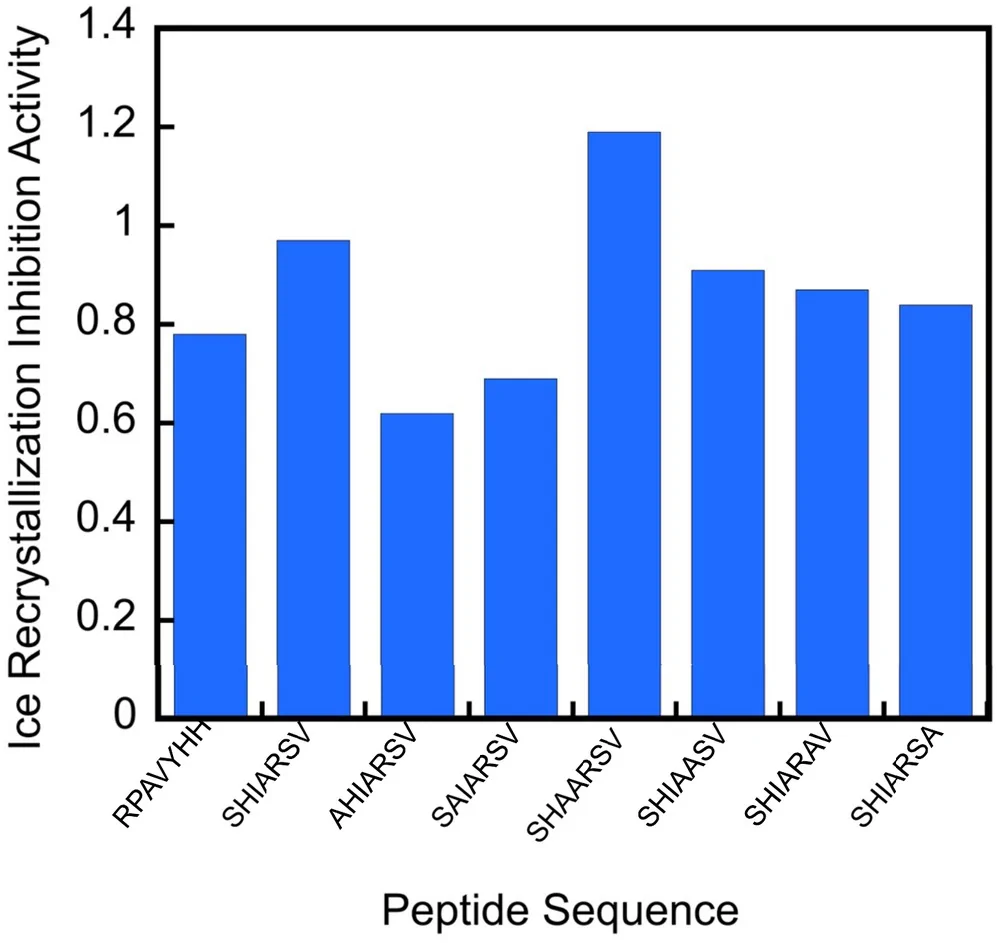
Ice recrystallization inhibition activity of RP and SH peptide analogs. The lower the IRI activity value is below 1.0, the higher the activity. The IRI activity value indicates that anti‐nucleating peptide controls the direction of ice growth by adsorbing onto ice nuclei, resulting in the formation of ice crystals with a small surface area and angular shapes.

In Figure 3, the lower the IRI activity value is below 1.0, the higher the activity. The IRI activity value indicates that anti‐nucleating peptides control the direction of ice growth by adsorbing onto ice nuclei, resulting in the formation of ice crystals with a small surface area and angular shapes.

It was found that, compared with the control (I), AHIARSV (C) produced the finest and most angular ice particles.

Figure 2 suggests that the ice crystals formed by SHAARSV, SHIARAV, and SHIARSA, in which the SH peptide was replaced with alanine are large, and it can be concluded that these peptides have low antifreeze activity. All the data suggest AHIARSV has high ANA and the ability to suppress ice recrystallization and is therefore considered to be the peptide with high antifreeze activity. In general, anti‐ice nucleating agents control the direction of ice growth by adsorbing onto ice nuclei, resulting in the formation of angular ice crystals. Based on the results in Figures 1 and 2, and judging from the ANA and RI activity values, we conclude that AHIARSV exhibits the highest activity as an anti‐nucleation agent.

It can be inferred that seven‐residue peptides do not form a specific conformation [50, 51, 52]. Therefore, it is thought that the AHIARSV and SHIARAV peptides interact with ice nuclei and exhibit anti‐ice‐nucleation activity.

Although it was only the Ser residues at positions 1 and 6 of SH peptide that were substituted separately, the results for ANA and IRI activity changed significantly; this suggests that the balance between hydrophobicity and hydrophilicity determined by the position of Ser within the peptide is important [53, 54]. It is expected that, by modifying the peptide sequence while considering the overall balance of the peptide, we will be able to identify peptides with antifreeze activity comparable with that of AFP.

## 4 Conclusions

Linear phage biopanning was performed through Round 4 using target of AgI. After confirming that the extracted DNA reached the required concentration, peptide sequences were analyzed, and the five type peptides were obtained. Among these, SH peptide exhibited the strongest anti‐ice‐nucleation activity. Three peptides, GASTRVQ, GAPNAVP, and QLVTGPS, could not be evaluated because they were insoluble or poorly soluble in water. To identify residues important for AgI binding and ANA, an alanine scan of SHIARSV was conducted, generating six variants: AHIARSV, SAIARSV, SHAARSV, SHIAASV, SHIARAV, and SHIARSA. No concentration‐dependent freezing‐point depression was observed, confirming that activity was not colligative. AHIARSV showed activity similar to SH peptide at 1.0 mg/mL but significantly higher activity at 2.0 and 4.0 mg/mL. When tested at 8.0 mg/mL, activity decreased sharply, indicating that the enhanced activity at intermediate concentrations was due to true anti‐nucleation effects rather than freezing‐point depression. SAIARSV exhibited reduced activity overall, especially at 1.0 mg/mL, suggesting that His [2] contributes to activity at low concentrations. SHAARSV showed no change compared with SH peptide, indicating that Ile [3] is not essential. SHIAASV displayed increased activity at 1.0 and 2.0 mg/mL but decreased activity at 4.0 mg/mL, implying a role for Arg [5] in stabilizing peptide–AgI interactions. SHIARAV and SHIARSA showed unusual decreases only at 2.0 mg/mL, suggesting that subtle hydrophobic/hydrophilic balance and residue positioning influence peptide–AgI interactions.

IRI activity was evaluated for all peptides. AHIARSV produced the smallest and most angular ice crystals, indicating strong suppression of recrystallization and confirming its high antifreeze activity. In contrast, SHAARSV, SHIARAV, and SHIARSA formed large ice crystals, demonstrating low antifreeze activity. The differing effects of substituting Ser at positions 1 and 6 highlight the importance of residue position in determining hydrophobic/hydrophilic balance and overall peptide function. These findings suggest that rational sequence modification considering this balance may lead to peptides with antifreeze activity comparable with natural antifreeze proteins.

## Conflicts of Interest

The authors declare no conflicts of interest.

## Data Availability

The data that support the findings of this study are available from the corresponding author upon reasonable request.
